# Preoperative PDW levels predict pulmonary metastasis in patients with hepatocellular carcinoma

**DOI:** 10.1186/s12885-022-09754-3

**Published:** 2022-06-21

**Authors:** Wen-juan Huang, Guang-yu Wang, Zeng-yao Liu, Meng-lin Zhang, Wen Wang, Xin Zhang, Rui-tao Wang

**Affiliations:** 1 Department of Internal Medicine, Harbin Medical University Cancer Hospital, Harbin Medical University, Harbin, Heilongjiang, 150081 China; 2 Department of Digestive Internal Medicine, Harbin Medical University Cancer Hospital, Harbin Medical University, Harbin, Heilongjiang, 150081 China; 3 Department of Interventional Medicine, The First Affiliated Hospital, Harbin Medical University, Harbin, Heilongjiang, 150001 China

**Keywords:** Hepatocellular carcinoma, Pulmonary metastases, Platelet distribution width, Prognosis

## Abstract

**Background:**

In hepatocellular carcinoma (HCC), pulmonary metastasis (PM) after hepatectomy is associated with poor clinical outcomes. The crucial phases of tumour cell proliferation, angiogenesis, and metastasis all entail platelet activation. In HCC, platelet distribution width (PDW) suggests platelet size changes and predicts a worse prognosis. The aim of this study was to assess the association between PDW and PMs in HCC patients receiving hepatectomy.

**Material/methods:**

From January 2013 to December 2015, a cohort of patients who underwent hepatectomy for HCC at the Harbin Medical University Cancer Hospital in China were retrospectively evaluated. The relationship between PDW levels and clinical and demographic parameters was examined. To investigate the relationships between predicted factors and PM, a competing risk model was used. From January 2016 to December 2018, a validation cohort of 109 patients from the First Affiliated Hospital of Harbin Medical University was studied independently.

**Results:**

In the primary cohort, 19 out of 214 patients had postoperative PMs. In HCC patients with PM, PDW levels were lower than in those without PM. There was a significant difference in the cumulative incidence of 2-year PM between the high-PDW and low-PDW groups after controlling for competing risk events (death prior to the development of PM) (p < 0.001). In addition, PDW was also found to be an independent predictor for PM in a multivariable competing risk analysis. The results were externally validated in another cohort.

**Conclusions:**

In HCC, preoperative PDW is significantly associated with PM. PDW could be a biomarker for post-operative PM in HCC patients.

## Introduction

Hepatocellular carcinoma (HCC) is the third most common cause of malignancy-related death [1, 2]. Curative hepatectomy remains the most common treatment for HCC patients. However, there is a substantial risk of recurrence after curative hepatectomy. Despite the fact that intrahepatic recurrence is more common, extrahepatic metastases (EHMs) represent 14.0% to 25.5% of all recurrences, After curative hepatectomy, pulmonary metastasis (PM) accounts for roughly half of all EHMs [3, 4].

The presence of PM after hepatectomy indicates a poor prognosis. Despite advances in PM therapy, strategies for accurately predicting the incidence of PM following curative hepatectomy remain inadequate [4]. Identifying high-risk patients for PM before surgery is helpful in early detection and early intervention. Thus, investigation of novel biomarkers for PM is urgently needed.

Recently, abundant evidence shows that platelet activation is involved in tumor proliferation, angiogenesis, and metastasis [5]. Through direct signal transduction with hepatocytes and liver parenchymal cells, platelets have been demonstrated to increase HCC cell proliferation and infiltration, as well as liver regeneration. Moreover, antiplatelet therapy has also been demonstrated to reduce liver damage and improve patient outcomes [6]. Platelet distribution width (PDW) reveals variations in platelet size and is considered a hallmark of platelet morphology [7, 8]. At present, PDW has been proven to be critical in the prediction of liver metastasis in colorectal cancer and distant metastasis in gastric cancer [9]. PDW levels were also found to predict poor survival in HCC in our previous study [10]. However, no study has investigated the association between PDW and PM following hepatectomy for HCC.

The aim of this study was to assess the association between PDW and PMs in HCC patients receiving hepatectomy.

## Materials and methods

### Patients

The clinical data of 214 patients with histologically diagnosed HCC at the Harbin Medical University Cancer Hospital in China were reviewed retrospectively from January 2013 to December 2015. All of the patients were subjected to radical surgical resection. They exhibited no signs of substantial portal vein/hepatic vein invasion and had not received any adjuvant therapy prior to surgery. This study excluded participants with other malignancies, haematological illness, infectious disease, and cardiovascular disease. The subjects who had treatment with anticoagulants, statins, or acetylic salicylic acid were also excluded. Information from another independent cohort of patients who underwent hepatectomy for HCC at the First Affiliated Hospital of Harbin Medical University, from January 2016 to December 2018, was retrospectively collected. Two hospitals' ethics committees gave their approval for the study. An informed consent form was signed by all participants.

### Data collection

The following demographic and clinicopathological information were collected: age, sex, body mass index (BMI), hepatitis B virus surface antigen (HBsAg), antibodies to hepatitis C virus (anti-HCV), the presence of liver cirrhosis, Child–Pugh’s grade, aspartate aminotransferase (AST), alanine aminotransferase (ALT), γ-glutamyl transferase (γ-GGT), albumin, total bilirubin, creatinine, alphafetoprotein (AFP), international normalized ratio (INR), tumor size, tumor number, capsule, tumor differentiation, macrovascular invasion, model of end-stage liver disease score (MELD score), fibrosis-4 index (FIB-4), aspartate aminotransferase to platelet ratio index (APRI), platelet-albumin-bilirubin (PALBI), and albumin-bilirubin (ALBI) score. White blood cell count (WBC), platelet count, PDW, mean platelet volume (MPV), and haemoglobin were directly obtained by an automated hematological analyzer. PDW had a normal range of 11–17%.

### Follow-up

After hepatectomy, patients were followed up every three months. At each appointment, a routine abdomen and chest computed tomography (CT) was conducted.

Liver function and serum AFP were measured. When tumor recurrence or metastasis is suspected, abdominal contrast-enhanced CT and/or magnetic resonance imaging (MRI) are performed every 6 months or earlier. The following criteria were used to diagnose PM: (a) A dynamic chest CT scan revealed freshly emerging lesions, particularly many round nodules around the lungs; and (b) AFP levels were increased. The CT findings were confirmed by at least two independent radiologists. Response Evaluation Criteria in Solid Tumors (RECIST) (version 1.1) was used to define the response. A bronchial perfusate examination and sputum cytological test were used to differentiate other pulmonary lesions. All of the patients were tracked for up to two years. Patients who were diagnosed with PM less than a month after hepatectomy were excluded.

### Statistical analysis

Statistical analyses were completed using SPSS software (version 26.0), and R software (version 4.1.2). Categorical and continuous variables were analyzed using Chi-squared test and Student’s *t*-test, respectively. Receiver operating characteristic (ROC) curve was constructed to define the optimal cut-off value of PDW using MedCalc software (version 15.0). Among these survival outcomes, PM was the interest event, while death was considered a competing risk. Survival analyses were performed using univariable and multivariable competing risk models. Variables were included in the multivariable competing risk analysis if the P value on univariable competing risk analysis was < 0.10. The cumulative incidence of PM was estimated using the cumulative incidence function (CIF) curves and intergroup comparison was analyzed using the Gray’s test. The results were presented as subdistribution hazard ratios (sHR) with 95% CI. *P* < 0.05 was regarded as significant.

### Results

The clinicopathological characteristics of HCC patients in the derivation set and validation set are summarized in Table 1. There were 214 patients (mean age, 52.6 ± 9.2 years; range, 26.0 to 74.0 years) in the derivation cohort, including 162 males (75.7%) and 52 females (24.3%). However, no statistically significant differences were detected between the derivation and validation cohorts with regard to age, sex, hepatitis C, vascular invasion, tumor number, MELD score, APRI score, FIB-4 score, AFP levels, platelet count, and ALT levels.

**Table 1:** Baseline characteristics of patients with HCC

Variables	Derivation set	Validation set	*P*-value
*N*	214	109	
Age (years)	52.6 ± 9.2	52.7 ± 10.1	0.966
BMI (kg/m^2^)	24.2 ± 3.8	22.0 ± 2.6	< 0.001
Sex (male, %)	162 (75.7)	82 (75.2)	0.926
HBsAg (%)	190 (88.8)	65 (59.6)	< 0.001
Hepatitis C (%)	11 (5.1)	2 (1.8)	0.153
Cirrhosis (%)	196 (91.6)	41 (37.6)	< 0.001
Child Pugh score			< 0.001
A	205 (95.8)	85 (78.0)	
B	9 (4.2)	24 (22.0)	
Vascular invasion			0.631
No	186 (86.9)	24 (22.0)	
Yes	28 (13.1)	85 (78.0)	
Tumor number			0.631
Single	183 (85.5)	91 (83.5)	
Multiple	31 (14.5)	18 (16.5)	
Tumor differentiation			< 0.001
Poor	38 (17.8)	50 (45.9)	
Moderate/well	176 (82.2)	59 (54.1)	
Capsule			0.001
Complete	183 (85.5)	77 (70.6)	
Incomplete	31 (14.5)	32 (29.4)	
Tumor size (cm)	4.9 ± 2.9	6.3 ± 4.2	0.002
MELD score	-1.84 (-3.58 to 0.02)	1.57 (-4.20 to 0.90)	0.451
FIB-4	1.9 (1.4–3.2)	1.9 (1.1–3.2)	0.417
APRI	0.6 (0.4–0.9)	0.7 (0.4–1.2)	0.439
PALBI	-3.3 (-3.6 to -3.1)	-4.8 (-5.2 to -4.2)	< 0.001
ALBI	-2.6 (-2.9 to -2.3)	-2.0 (-2.4 to -1.7)	< 0.001
AFP (ng/mL)	14.8 (4.1–227.5)	23.7 (4.7–471.2)	0.390
WBC (× 10^9^/L)	5.84 ± 2.10	8.39 ± 3.98	< 0.001
Haemoglobin (g/L)	142.0 ± 17.3	122.2 ± 23.7	< 0.001
Platelet count (× 10^9^/L)	161.5 ± 63.2	177.9 ± 90.6	0.093
MPV (fL)	10.6 ± 1.4	11.1 ± 1.1	< 0.001
PDW (%)	15.2 ± 2.4	13.2 ± 2.2	< 0.001
INR	1.06 ± 0.09	1.21 ± 0.26	< 0.001
Albumin (g/L)	39.5 ± 4.6	34.3 ± 5.8	< 0.001
Creatinine (μmol/L)	76.8 ± 15.0	60.6 ± 15.5	< 0.001
AST (U/L)	35 (28–47)	51 (31–88)	< 0.001
ALT (U/L)	39 (28–52)	39 (26–60)	0.589
γ-GGT (U/L)	49 (32–84)	78 (44–135)	< 0.001
Total bilirubin (μmol/L)	13.9 (10.4–18.7)	18.2 (14.1–25.6)	< 0.001

AFP Alphafetoprotein, *ALB*I Albumin-bilirubin, *APRI* Aspartate aminotransferase to platelet count ratio index, *AST* Aspartate aminotransferase, *ALT* Alanine aminotransferase, *BMI* Body mass index, *FIB-4* Fibrosis-4 index, *γ-GGT* γ-glutamyl transferase, *INR* International normalized ratio, *MPV* Mean platelet volume, *MELD score* model of end-stage liver disease score, *PDW*, Platelet distribution width, *PALBI* Platelet-albumin-bilirubin, *WBC* White blood cell

Table 2 displays the characteristics of HCC patients stratified by PM status. In the derivation cohort, over a median follow-up period of 27.0 (range 4.0–82.0) months, 19 (8.88%) patients had PM events. Moreover, statistical significance was found in vascular invasion, capsule, tumor size, AFP, and PDW levels between the PM and non-PM groups (Table 2). Other clinical parameters were not in correlation with PM. In the validation set, the median follow-up time was 25.0 (range 4.0–46.0) months. Cirrhosis, Child Pugh score, tumor size, MELD score, PALBI, AFP, haemoglobin, platelet count, and PDW levels between the two groups had a significant difference.

**Table 2:** The characteristics of HCC patients stratified by PM status

Variables	Without PM	With PM	*P*-value
**Development set**			
*N*	195	19	
Age (years)	52.6 ± 9.2	53.0 ± 9.1	0.855
BMI (kg/m^2^)	24.2 ± 3.8	24.4 ± 3.5	0.780
Sex (male, %)	151 (77.4)	11 (57.9)	0.058
HBsAg (%)	173 (88.7)	17 (89.5)	0.921
Hepatitis C (%)	10 (5.1)	1 (5.3)	0.980
Cirrhosis (%)	179 (91.8)	17 (89.5)	0.728
Child Pugh score			0.810
A	187 (95.9)	18 (94.7)	
B	8 (4.1)	1 (5.3)	
Vascular invasion			< 0.001
No	176 (90.3)	10 (52.6)	
Yes	19 (9.7)	9 (47.4)	
Tumor number			0.060
Single	164 (84.1)	19 (100.0)	
Multiple	31 (15.9)	0 (0.0)	
Tumor differentiation			0.099
Poor	32 (16.4)	6 (31.6)	
Moderate/well	163 (83.6)	13 (68.4)	
Capsule			0.004
Complete	171 (87.7)	12 (63.2)	
Incomplete	24 (12.3)	7 (36.8)	
Tumor size (cm)	4.7 ± 2.8	7.5 ± 3.5	< 0.001
MELD score	-1.8 (-3.6 to -0.0)	-2.0 (-2.9 to 1.2)	0.422
FIB-4	1.9 (1.3–3.1)	2.3 (1.6–3.3)	0.327
APRI	0.6 (0.4–0.9)	0.7 (0.4–0.9)	0.714
PALBI	-3.3 (-3.6 to -3.0)	-3.3 (-3.7 to -3.2)	0.353
ALBI	-2.6 (-2.9 to -2.3)	-2.7 (-3.0 to -2.4)	0.201
AFP (ng/mL)	12.3 (3.9–174.3)	231.0 (22.6–11,894.3)	0.001
WBC (× 10^9^/L)	5.82 ± 1.92	6.06 ± 3.48	0.634
Haemoglobin (g/L)	142.5 ± 16.3	137.4 ± 25.7	0.404
Platelet count (× 10^9^/L)	160.3 ± 64.3	174.0 ± 50.5	0.367
MPV (fL)	10.7 ± 1.5	10.2 ± 1.1	0.175
PDW (%)	15.4 ± 2.3	13.3 ± 2.3	< 0.001
INR	1.05 ± 0.08	1.08 ± 0.17	0.255
Albumin (g/L)	39.4 ± 4.6	40.9 ± 4.7	0.158
AST (U/L)	35 (28–47)	38 (34–56)	0.137
ALT (U/L)	40 (28–53)	31 (26–50)	0.250
γ-GGT (U/L)	49 (32–84)	48 (34–76)	0.927
Total bilirubin (μmol/L)	13.9 (10.4–18.7)	14.6 (10.4–21.2)	0.423
Creatinine (μmol/L)	76.8 ± 15.1	76.6 ± 14.2	0.967
**Validation set**			
*N*	98	11	
Age (years)	53.2 ± 9.8	47.9 ± 11.9	0.099
BMI (kg/m^2^)	22.1 ± 2.6	21.0 ± 2.3	0.193
Sex (male, %)	76 (77.6)	6 (54.5)	0.094
HBsAg (%)	60 (61.2)	5 (45.5)	0.312
Hepatitis C (%)	2 (2.0)	0 (0)	0.632
Cirrhosis (%)	40 (40.8)	1 (9.1)	0.039
Child Pugh score			0.048
A	79 (80.6)	6 (54.5)	
B	19 (19.4)	5 (45.5)	
Vascular invasion			0.063
No	24 (24.5)	0 (0)	
Yes	74 (75.5)	11 (100.0)	
Tumor number			0.311
Single	83 (84.7)	8 (72.7)	
Multiple	15 (15.3)	3 (27.3)	
Tumor differentiation			0.212
Poor	43 (43.9)	7 (63.6)	
Moderate/well	55 (56.1)	4 (36.4)	
Capsule			0.591
Complete	70 (71.4)	7 (63.6)	
Incomplete	28 (28.6)	4 (36.4)	
Tumor size (cm)	5.6 ± 3.5	13.0 ± 3.4	< 0.001
MELD score	-1.4 (-4.0 to -1.3)	-4.3 (-6.3 to-1.6)	0.041
FIB-4	2.0 (1.3–3.3)	0.9 (0.4–3.1)	0.103
APRI	0.7 (0.4–1.2)	0.4 (0.2–1.2)	0.300
PALBI	-4.8 (-5.2 to -4.1)	-5.3 (-5.8 to -4.7)	0.050
ALBI	-2.0 (-2.4 to -1.7)	-2.0 (-2.5 to -1.8)	0.904
AFP (ng/mL)	16.2 (4.5–416.4)	415.7 (94.4–1000.0)	0.001
WBC (× 10^9^/L)	8.45 ± 4.08	7.80 ± 2.98	0.609
Haemoglobin (g/L)	123.9 ± 22.3	107.0 ± 31.3	0.024
Platelet count (× 10^9^/L)	171.8 ± 87.0	232.3 ± 108.3	0.035
MPV (fL)	11.2 ± 1.1	10.7 ± 0.5	0.134
PDW (%)	13.4 ± 2.1	11.2 ± 1.1	< 0.001
INR	1.21 ± 0.27	1.17 ± 0.11	0.613
Albumin (g/L)	34.4 ± 5.9	33.6 ± 5.9	0.639
Creatinine (μmol/L)	60.8 ± 13.9	59.7 ± 27.0	0.902
AST (U/L)	38 (27–57)	56 (24–73)	0.794
ALT (U/L)	52 (31–87)	50 (26–203)	0.665
γ-GGT (U/L)	78 (42–134)	77 (65–252)	0.289
Total bilirubin (μmol/L)	18.9 (14.6–26.0)	14.0 (10.4–25.4)	0.155

Abbreviations: see to Table 1

**Fig. 1:**
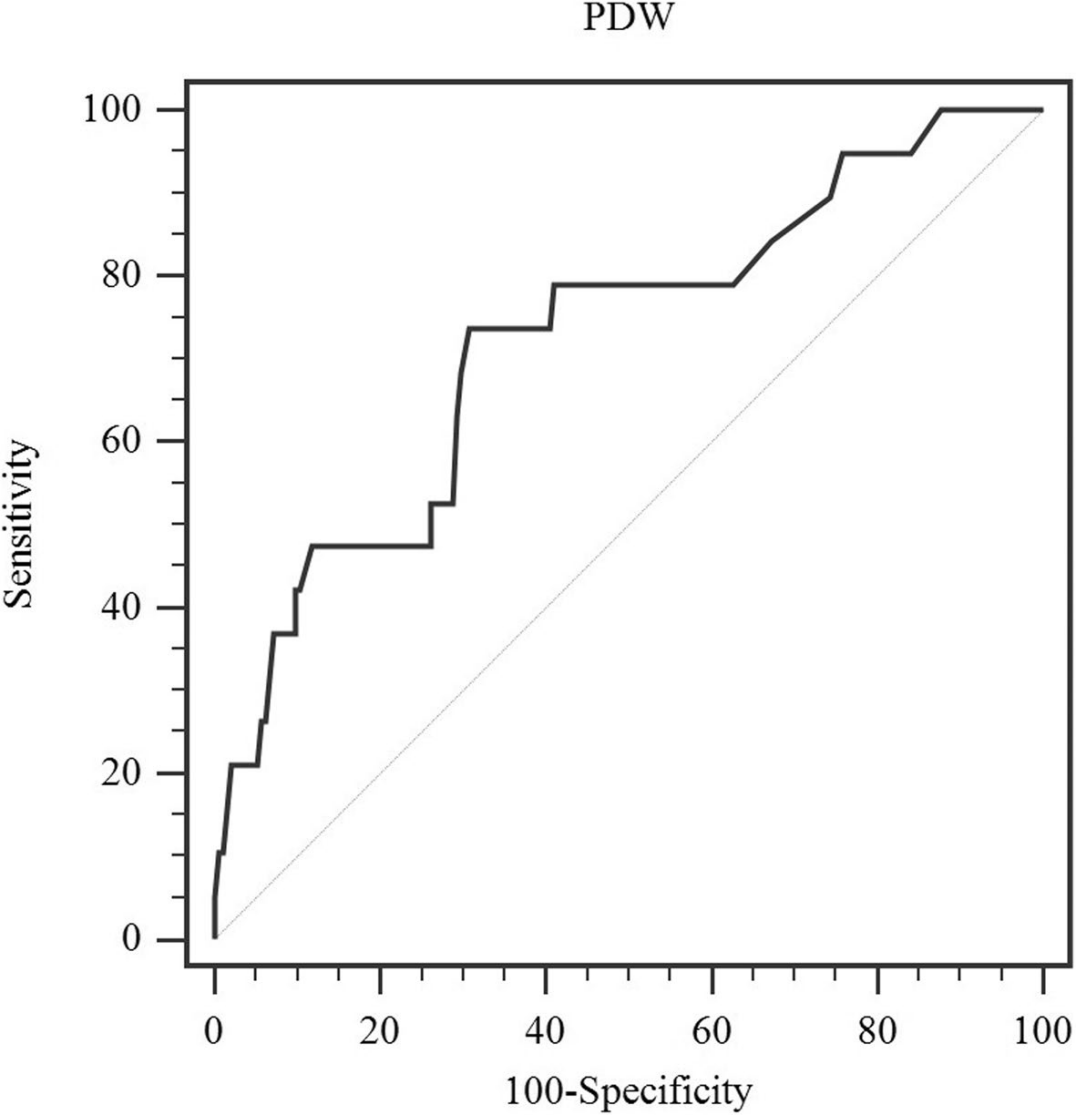
ROC curve to determine an optimal cut-off value of PDW

The optimal cut-off value of PDW was determined as 14.1% with an area under the curve (AUC) value of 0.732 (0.667–0.790) using ROC curve in the derivation cohort (Fig. 1). The HCC patients were classified into two parts based on the cut-off value (low-PDW (≤ 14.1%) and high-PDW (> 14.1%)). Among all patients, 57 patients (26.6%) had PDW ≤ 14.1 and 157 patients (73.4%) had PDW > 14.1. Over a median follow-up of 27.0 months, 7 patients in high-PDW group and 12 patients in low-PDW group had PM events. In the validation cohort, the HCC patients were classified into two parts by the same cut-off value of PDW. During the follow-up of 25.0 months, 1 patient in high-PDW group and 10 patients in low-PDW group developed PM.

Death was treated as an event competing with PM. Table 3 shows the results of the competing-risk analysis. In the univariable analysis, sex, vascular invasion, capsule, PDW (continuous variable) and tumor size (continuous variable) were significant prognostic factors for PM in the derivation cohort (p < 0.05). All these factors were included in the multivariable model. The multivariable competing-risk analysis revealed that PDW (sHR = 0.850, 95%CI [0.736–0.983]) and tumor size (sHR = 1.240, 95%CI [1.045–1.481]) were the independent predictive factors for PM. The same results were externally validated in another cohort.

**Table 3:** The predictors of PM in HCC patients

Variables	**Univariable**		**Multivariable**	
	**sHR (95% CI)**	***P*-value**	**sHR (95% CI)**	***P*-value**
**Development set**				
Sex (male vs female)	0.405(0.164–0.997)	0.049	0.443(0.170–1.153)	0.095
Vascular invasion (Yes vs No)	5.360(2.200–13.100)	< 0.001	2.610(0.898–7.596)	0.078
Tumor number (Multiple vs Single)	0.712(0.209–2.430)	0.590		
Tumor differentiation (Poor vs Moderate/well)	1.310(0.498–3.450)	0.580		
Capsule (Incomplete vs Complete)	3.110(1.230–7.850)	0.017	1.490(0.534–4.181)	0.440
Tumor size (cm)	1.330(1.200–1.460)	< 0.001	1.240(1.045–1.481)	0.014
AFP (ng/mL)	1.000(1.000–1.000)	0.090		
PDW (%)	0.798(0.706–0.901)	< 0.001	0.850(0.736–0.983)	0.028
**Validation set**				
Cirrhosis (Yes vs No)	0.089(0.011–0.703)	0.022	0.376(0.028–4.970)	0.460
Child Pugh score (B vs A)	6.270 (1.970–19.900)	0.001	2.587(0.378–17.682)	0.330
Tumor size (cm)	1.530(1.350–1.730)	< 0.001	1.940(1.291–2.915)	0.001
MELD score	0.944(0.686–1.300)	0.720		
PALBI	0.400(0.146–1.100)	0.070		
AFP (ng/mL)	1.000(1.000–1.000)	0.630		
Haemoglobin (g/L)	0.972(0.961–0.983)	< 0.001	1.046(0.988–1.107)	0.120
Platelet count (× 10^9^/L)	1.010(1.000–1.010)	0.016	1.001(0.990–1.011)	0.920
PDW (%)	0.764(0.645–0.904)	0.001	0.523(0.304–0.898)	0.019

sHR Subdistribution hazard ratio, *CI* Confidence interval Abbreviations: see to Table 1

**Fig. 2:**
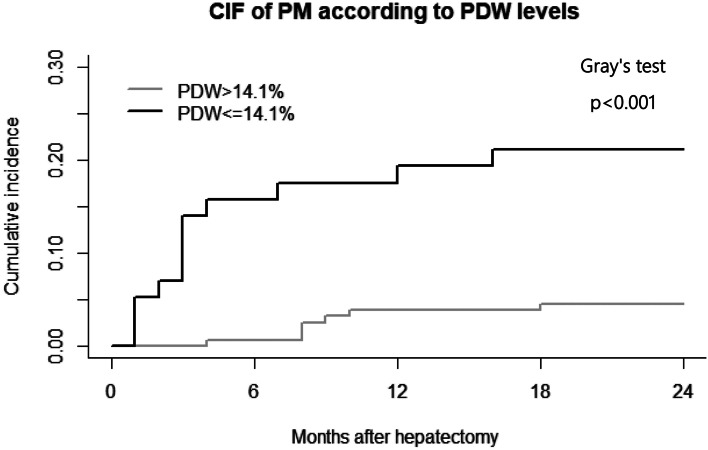
Timing of development of pulmonary metastases in development set

**Fig. 3:**
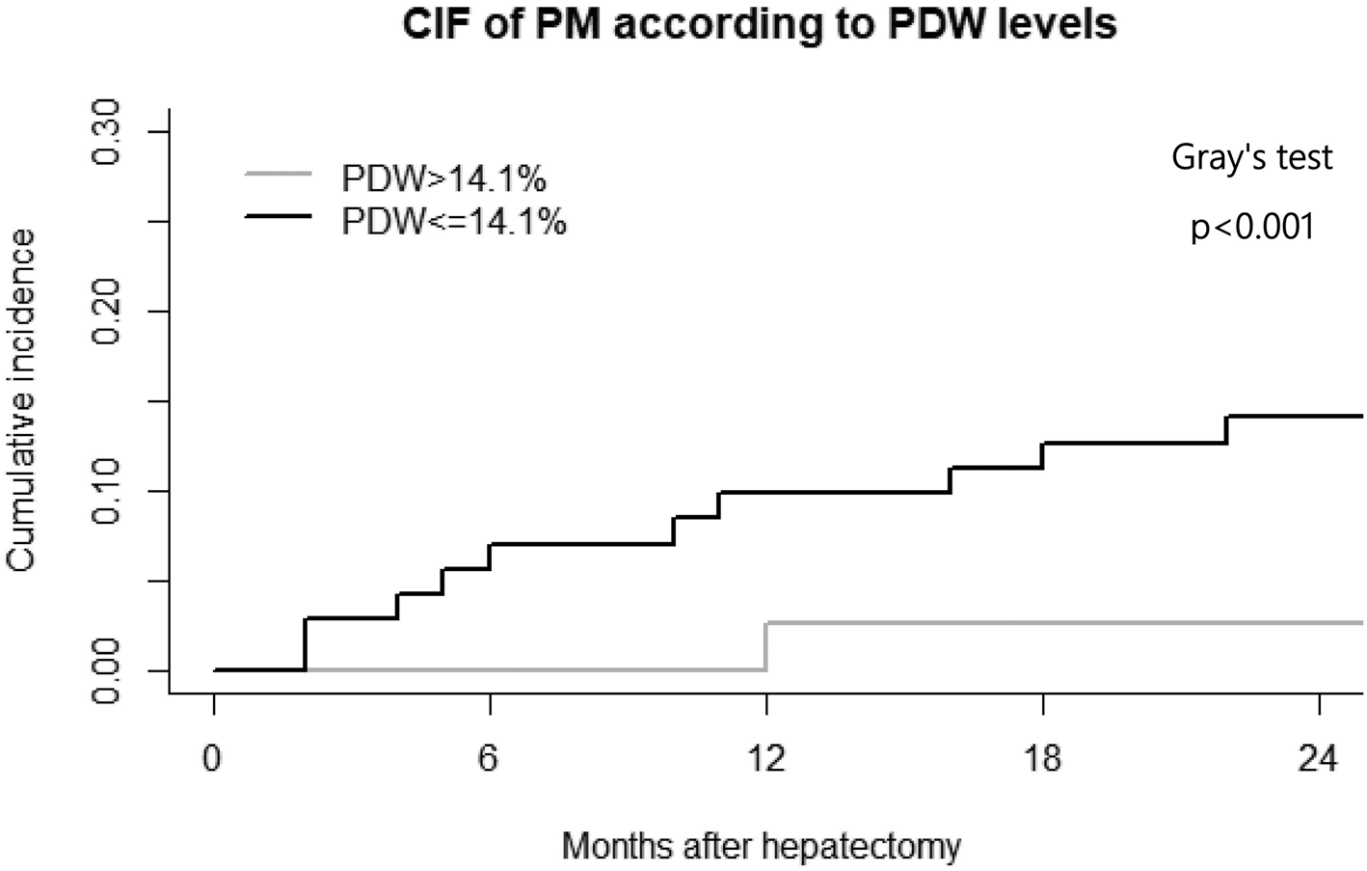
Timing of development of pulmonary metastases in validation set

In the derivation cohort, Fig. 2 showed the cumulative incidence of PMs in the high-PDW and low-PDW groups. PM was the interest event, while death was considered a competing risk. After controlling for competing risk event, there was a significant difference in the incidence of PM between the high-PDW and low-PDW groups (*p* < 0.001). We found that individuals with low PDW levels tended to develop PM more than individuals with high PDW levels, with 2-year cumulative incidence of 21.0% and 4.5%, respectively. In the validation cohort, the cumulative incidence of 2-year PM in HCC patients was 21.3% in the high-PDW group and 1.6% in the low-PDW group (*p* < 0.001) (Fig. 3**)**.

## Discussion

This study observed that in HCC patients with PM, PDW levels were lower than in those without PM. In addition, multivariable analysis found that PDW was the independent predictor of PM after HCC resection. And an external validation cohort came to the same conclusion.

Platelets are traditionally considered the principal cells active in thrombosis and hemostasis. Extensive research has demonstrated that platelets make a substantial contribution to cancer growth and dissemination. Platelet activation is caused by tumor cells interacting with platelets, which promotes tumor development and metastasis [11]. Although the functions of platelets in tumor metastasis have been widely studied in other malignancies, the exact effects of platelets on HCC metastasis are unknown [12–14]. Compared with HCC patients without metastases, the patients with extrahepatic metastases had a higher platelet count. Moreover, platelet count is a valuable diagnostic for predicting extrahepatic metastasis in patients with early-stage HCC receiving curative therapy [15]. In a metastatic HCC mouse model, pharmacological inhibition of platelet activation prevents platelets from adhering to tumor cells and reduces metastasis [16]. Krüppel-like factor 6 (KLF6), a tumor suppressive gene, inhibits tumor growth and invasion in HCC. Previous studies revealed that platelet release downregulates KLF6 expression in vivo and in vitro in HCC cells [17]. In addition, platelet extracts may also be able to counteract sorafenib or regorafenib-mediated inhibitory effects in HCC cells [18]. According to our findings, platelet activation has a crucial role in HCC. Furthermore, our data supports the use of antiplatelet treatment in patients with HCC who have undergone hepatectomy.

The mechanisms behind the link between decreased PDW and PMs are still unknown. PDW is an early biomarker of platelet activation and indicates the average change in platelet volume. In megakaryocyte development and thrombopoiesis, platelet volume is determined. The failure of heterogenic megakaryocytic maturation is reflected in the decline in PDW levels [19]. In addition, numerous clinical studies revealed a strong link between PDW and the prognosis of various cancers such as breast cancer, colon cancer, ovarian cancer, and non-small cell lung cancer [20–23]. Meanwhile, several reports have also confirmed that PDW is an independent predictor of poor clinical outcome in HCC [10, 24, 25]. Interleukin-6 (IL-6), granulocyte colony-stimulating factor (G-CSF), and macrophage colony-stimulating factor (M-CSF) have all been found to influence megakaryocytic maturation, platelet production, and platelet volume [26]. Furthermore, tumor-derived G-CSF creates a pre-metastatic environment in distant organs, and anti-G-CSF or anti-M-CSF antibodies have been shown to significantly prevent PMs [27]. Furthermore, the presence of thrombocytopenia in HCC patients with cirrhosis indicates that the disease is in an advanced stage. Thrombocytopenia before treatment could be a low-cost and practical predictor of postoperative recurrence in HCC patients [28]. This also partly explains why PDW levels in HCC patients with PM were lower than those without PM.

In the present study, there are several limitations that deserve mention. Firstly, it was a small-sized study with a retrospective nature. Secondly, the mechanisms of PDW involved in PM were not explored and further research is needed. Lastly, participants only included Chinese people, so a larger study is needed to extrapolate our findings to other ethnic groups.

In brief, preoperative PDW may predict PM in HCC patients. Further studies are warranted.

## Data Availability

The data used in the study can be obtained from the corresponding author upon request.

## Abbreviations

AFP: Alphafetoprotein
AST: Aspartate aminotransferase
ALT: Alanine aminotransferase
APRI: Aspartate aminotransferase to platelet count ratio index
BMI: Body mass index
CIF: Cumulative incidence function
CT: Computerized tomography
CI: Confidence interval
EHMs: Extrahepatic metastases
FIB-4: Fibrosis-4 index
γ-GGT: γ-Glutamyl transferase
G-CSF: Granulocytes colony-stimulating factor
HCC: Hepatocellular carcinoma
IL-6: Interleukin-6
INR: International normalized ratio
IQR: 25%, 75% Interquartile range
KLF6: Krüppel-like factor 6
MPV: Mean platelet volume
M-CSF: Macrophage colony-stimulating factor
MELD score: Model of end-stage liver disease score
PALBI: Platelet-albumin-bilirubin
ALBI: Albumin-bilirubin
PM: Pulmonary metastasis
PDW: Platelet distribution width
RECIST: Response evaluation criteria in solid tumors
ROC: Receiver operating characteristic
sHR: Subdistribution hazard ratios
SD: Standard deviation
WBC: White blood cell
